# Application of Salivary Alpha-1 Antitrypsin in the Diagnosis of Rheumatoid Arthritis: A Pilot Study

**DOI:** 10.3390/medicina60040605

**Published:** 2024-04-06

**Authors:** Sang Wan Chung, Seung-Jae Hong

**Affiliations:** Division of Rheumatology, Department of Internal Medicine Kyung Hee University College of Medicine, Kyung Hee University Medical Center, Seoul 02447, Republic of Korea

**Keywords:** rheumatoid arthritis, seronegative rheumatoid arthritis, salivary *α*1-Antitrypsin, salivary biomarker

## Abstract

*Background and Objective:* Rheumatoid arthritis (RA) is an autoimmune disease in which joints are gradually destroyed. Early diagnosis and treatment before joint deformation or destruction is important. The detection of novel RA biomarkers in saliva may facilitate early detection of RA before disease onset. This study aimed to evaluate salivary concentration of *α*1-antitrypsin (A1AT) in healthy patients and those with RA, and to assess the diagnostic value of salivary A1AT. *Materials and Methods:* In total, 80 participants were included: 20 healthy participants, and 60 patients with RA. Saliva and serum samples were obtained from all the patients. Levels of A1AT and cytokines, including interleukin-1 beta (IL-1*β*), IL-6, and IL-10 in saliva and serum, were evaluated using an enzyme-linked immunosorbent assay kit and Luminex assay. Data were analyzed using SPSS for Windows. *Results:* There was a higher level of A1AT in the saliva of patients with RA (median: 2388.66 ng/mL) than that in healthy controls (1579.06 ng/mL). There was a positive mild-to-moderate accuracy (area under the curve: 0.57–0.85) of A1AT in saliva to diagnose RA. The cut-off level (ng/mL) of A1AT in saliva for detecting RA was 1689.0. *Conclusions:* The obtained data can promote the application of the measurements of A1AT in saliva to diagnose RA.

## 1. Introduction

Rheumatoid arthritis (RA) is a chronic inflammatory autoimmune disease characterized by arthritis. Rheumatoid arthritis exhibits several extra-articular manifestations that result in joint destruction and are associated with progressive disability [1]. It is well known that early diagnosis and early treatment of RA are important in preventing such joint deformation [2]. The current American College of Rheumatology (ACR)/European League Against Rheumatism (EULAR) 2010 classification criteria for RA included two serum autoantibodies: rheumatoid factor (RF) and anti-citrullinated protein antibodies (ACPA) [3]. Nevertheless, 15–25% of patients may present with clinical symptoms suggestive of RA but remain persistently negative on conventional RA immunological tests. Additionally, 30–45% of patients have negative RF during early stages of RA [4]. Therefore, there is an urgent need to identify new biomarkers for the diagnosis of RA.

Serine protease inhibitors are anti-inflammatory reactants, and alpha-1 antitrypsin (A1AT), a protease inhibitor derived from serum, is enhanced by inflammatory cytokines and endotoxins. A1AT is mainly produced by hepatocytes and, to a lesser extent, expressed by other cell types, such as monocyte-derived macrophages and dendritic cells, alveolar macrophages, pancreas, enterocytes, the endothelium, activated neutrophils, and some cancer cells [5]. The main function of inhibiting enzymes released during injury and inflammation is to prevent tissue damage caused by protease overactivation [6]. Multiple studies have found the unique role of A1AT in anti-inflammatory and immunomodulatory activity, which are independent of its anti-protease activity. In healthy individuals, the plasma level of A1AT is 0.9–2 g/L, which will increase about 4 to 5 times during acute inflammation or infection [7]. Autoimmune diseases including RA are closely related to excessive inflammation. To date, A1AT has shown beneficial effects on many autoimmune diseases, including rheumatoid arthritis, systemic lupus erythematosus, systemic sclerosis, diabetes, and acute graft versus host disease, in patients and animal models. Therefore, A1AT is also considered an inflammatory-related molecule [8,9]. Limited studies have previously investigated the level of A1AT in the serum of patients with RA [10,11,12]. However, only one study on salivary A1AT reported the diagnostic utility of A1AT in patients with seronegative RA [13]. The present study investigated the relationship between salivary cytokines and RA, and the diagnostic value of these salivary biomarkers, especially A1AT.

## 2. Methods

### 2.1. Study Design and Population

Serum samples from 60 patients with RA and 20 healthy controls (HC) were collected for biomarker identification from the Kyung Hee University Hospital. The Institutional Review Board approved this study (KHMC 2021-11-008). Written informed consent was obtained from all the participants before their inclusion in the study. Participants who were diagnosed by rheumatologists and fulfilled the 1987 ACR classification criteria for RA [14] or 2010 ACR/EULAR classification criteria for RA [3] were recruited as a patient group. Healthy controls without RA who visited a health checkup center were recruited. Sampling for A1AT in saliva and serum was performed simultaneously. Whole saliva was collected by spitting for 5 min before eating any food. Eating and drinking were stopped 30 min prior to taking saliva. Saliva was allowed to accumulate in the floor of the mouth, and the patient spat it into test tubes [15]. Salivary A1AT levels were quantified using a commercially available enzyme-linked immunosorbent assay kit (Abcam, Waltham, MA, USA). Serum and salivary cytokine levels, including those of interleukin-1 beta (IL-1 *β*), IL-6, and IL-10, were measured using a multiplex assay with Luminex bead technology (Luminex, Austin, TX, USA).

### 2.2. Clinical Variables

Age, sex, smoking status, RA duration, RF positivity, ACPA positivity, erythrocyte sedimentation rate (ESR), C-reactive protein (CRP) level, tender joint count, swollen joint count, Patient Global Assessment, and disease activity score of 28 joints (DAS28-ESR) at baseline were recorded by a medical chart review. Mean values of hematological indicators, including RF, ACPA, ESR, and CRP were measured in the serum of all patients with RA. The values were dichotomized; positive when above the threshold, negative when below the threshold. ACPA ≥ 20 IU/mL [16] and RF ≥ 20 IU/mL [17] https://www.ncbi.nlm.nih.gov/pmc/articles/PMC10881463/ (accessed on 6 February 2024) were considered positive.

### 2.3. Statistical Analysis

Differences between the two groups (RA and HC groups) were compared using descriptive statistics. Categorical variables were compared using the chi-square test or Fisher’s exact test, and continuous variables were compared using the Kruskal–Wallis test. A simple correlation test (Pearson’s correlation test) was used to analyze correlations between variables. Additionally, a multiple regression analysis was used to analyze the correlation between variables after adjusting for basic confounders, such as age and sex. A receiver operating characteristic (ROC) curve analysis was used to identify cut-off levels of A1AT for detecting the study outcome. Data are presented as mean ± standard deviation (SD) (for variables with normal distributions), median and interquartile range (for variables with skewed distributions), or subject number. All statistical calculations were performed with SPSS version 22 (SPSS Inc., Chicago, IL, USA), and the statistical significance level was set at a *p*-value of 0.05.

## 3. Result

As shown in Table 1, of the 60 patients with RA and 20 HC enrolled, 58 (72.5%) were females. The mean (SD) age was 56.58 (±12.49) years old in the RA group, and 43.85 (±7.87) years old in the HC group. In the RA group, 20 (33.3%) patients were seronegative for RA.

Significantly higher salivary levels of A1AT were found in the RA group when compared to those in the HC group (*p* = 0.021). Salivary IL-1*β*, IL-6, and IL-10 levels were higher in concentration in the RA group compared to those in the HC group, and the difference between the groups was not statistically significant (*p* = 0.690, *p* = 0.338, and *p* = 0.304, respectively) (Table 2).

**Figure 1:**
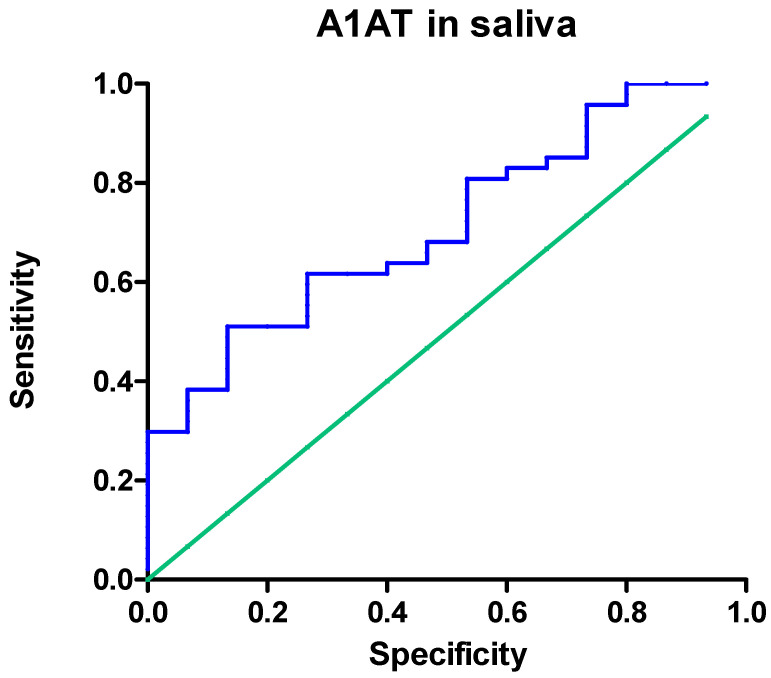
Receiver operating characteristic curve analysis of *α*1-antitrypsin in saliva, AUC of 0.7135, *p* = 0.0134; AUC, area under the curve; A1AT, *α*1-antitrypsin.

Table 3 and Figure 1 show the ROC curve analysis of A1AT in saliva. The area under the curve indicated a significantly moderate accuracy for patients with RA, and the cut-off value (ng/mL) for detecting RA was 1689.0 in the saliva.

In the sub-analysis of seronegative RA (Table 4), the level of salivary A1AT was higher than HC; however, this was not statistically significant.

## 4. Discussion

Rheumatoid arthritis is a debilitating disease characterized by joint inflammation, pain, and structural damage. Despite recent successes in therapeutic development, only early diagnosis and treatment are effective in preventing joint deformations. The current classification criteria for RA include two autoantibodies, RF and ACPA, and their key role in the diagnosis of the disease is emphasized [18]. There is a need to develop diagnostic biomarkers for early diagnosis of RA, including for patients with seronegative RA.

The relationship between the pathophysiology of RA and saliva is well-known. Several epidemiological studies have reported a correlation between RA and periodontitis [19,20]. In 2016, Fuggle et al. reported a meta-analysis of 21 studies and found that periodontitis was more frequent in patients with RA than in HCs (risk ratio 1.13) [21]. Periodontitis is associated with RA severity. Rheumatoid arthritis disease activity scores are higher in patients with RA who have more serum antibodies against *Porphyromonas gingivalis*, an oral anaerobe involved in the development of periodontitis [22]. For these reasons explained above, we focused on salivary biomarkers for the diagnosis of RA.

A1AT, a protease inhibitor derived from serum, is considered an inflammatory-related molecule. This study investigated the relationship between salivary A1AT and RA, and the diagnostic value of salivary A1AT. The present study is the first to clinically investigate the diagnostic value of salivary A1AT in RA using an enzyme immunoassay.

Alpha-1 antitrypsin, a member of the serine protease inhibitor family, was first isolated in 1955 and named after its ability to inhibit trypsin, with the highest affinity for neutrophil elastase [23,24]. Alpha-1 antitrypsin is mainly produced by hepatocytes and secreted into the blood, and A1AT concentrations increase to approximately four-fold during infection and inflammation [25]. Alpha-1 antitrypsin not only serves as a protease inhibitor but also shows an increasing number of functions, including anti-protease, anti-inflammatory, anti-oxidant, anti-apoptotic, anti-viral, and anti-bacterial properties, as well as effects on immune cells [26]. Overactivation of the immune system is a characteristic of the pathogenesis of autoimmune diseases. Studies on the role of A1AT in RA have shown that ADAMTS-4 colocalized with A1AT plays a key role in the wear of cartilage aggregates, and is associated with the onset of RA [27].

In the present study, a statistically significant increase in salivary A1AT levels was observed in the RA group. Furthermore, similar salivary A1AT values were observed in patients with seronegative RA compared to those in patients with seropositive RA. This may be helpful in terms of the diagnostic value of RA, even in patients with seronegative RA. We did not find any correlation between salivary A1AT and disease activity (DAS28) or serological parameters (ESR and CRP). A larger cohort of patients should be evaluated for better characterization.

In the sub-analysis of salivary cytokines, there were no differences in other salivary cytokines; however, serum IL-6 and IL-10 in the blood were significantly increased. This shows that IL-6 and IL-10 are cytokines related to inflammation, and the fact that they are increased in RA patients is consistent with the results of previous studies. Madhoc et al. reported that serum IL-6 levels in RA correlates with clinical and laboratory indices of disease activity [28]. IL-10 has been shown to exert both anti-inflammatory and immunostimulatory effects, and there is increased production of IL-10 by non-T cells in patients with RA [29]. In the case of salivary cytokines, the trend was consistent with previously published research results. Kaczyński et al. reported that salivary IL-6 levels in RA patients were not high. They explained that this might be related to DMARD or glucocorticosteroid therapy [30]. 

This study is meaningful in that it is the first to use salivary A1AT in the diagnosis of RA. However, the study was limited by its small sample size. Potential patients with RA were not completely excluded from the HC group because screening for RF and ACPA were not performed. Patients with arthritis were excluded, as much as possible, because they had no arthralgia during interviews. Future studies with larger sample sizes are required.

## 5. Conclusions

In conclusion, salivary A1AT is a potential diagnostic biomarker for RA. The measurement of this biomarker can be applied in clinical practice for RA, but more multifaceted studies are needed.

## Figures and Tables

**Table 1:** Clinical and demographic characteristics of enrolled patients.

	RA (*n* = 60)	HC (*n* = 20)	*p*-Value
**Age, year**	56.58 ± 12.49	43.85 ± 7.87	<0.001
**Sex**			0.161
**Male**	14 (23.3)	8 (23.3)	
**Female**	46 (76.7)	12 (60.0)	
**Saliva for 5 min (mL)**	3.47 ± 2.45	4.86 ± 2.37	0.035
**Comorbidities**			
HTN	16 (26.7)	4 (20.0)	0.392
Dyslipidemia	22 (36.7)	1 (5.0)	0.004
DM	5 (8.3)	1 (5.0)	0.530
COPD	6 (10.0)	0 (0)	0.167
Chronic liver disease	2 (3.3)	1 (3.8)	0.583
**Laboratory data**			
ESR (mm/h)	24.88 ± 19.77	-	
CRP (mg/dL)	0.59 ± 0.61	-	
RF positive	40 (66.7)	-	-
RF (IU/mL)	124.48 ± 212.88	-	
Anti-CCP positive,	40 (66.7)	-	-
Anti-CCP (units/mL)	268.82 ± 481.47	-	
Seronegative RA	20 (33.3)		
Anti Ro	3 (14.3)		
**DAS28-ESR**	2.71 ± 0.96	-	
**DAS28-CRP**	2.12 ± 2.49	-	

HTN, hypertension; DM, diabetes mellitus; COPD, chronic obstructive pulmonary disease; ESR, erythrocyte sedimentation rate; CRP, C-reactive protein; RF, rheumatoid factor; anti-CCP, anti-cyclic citrullinated peptide; RA, rheumatoid arthritis; HC, healthy control; DAS28, disease activity score of 28 joints.

**Table 2:** Salivary and serum levels of A1AT, IL-6, IL-1*β*, and IL-10 in patients with RA and healthy controls.

Average Concentration of Studied Cytokines	RA	HC	*p*-Value
**Salivary A1AT (ng/mL)**	2388.66 ± 1875.80	1579.06 ± 1067.06	0.021
**Serum A1AT (mg/mL)**	1.7879 ± 1.5933	1.5932 ± 3.7173	0.082
**Salivary IL-1*β***	540.89 ± 777.05	488.25 ± 379.03	0.690
**Serum IL-1*β***	1.5497 ± 0.1583	1.5780 ± 0.1388	0.451
**Salivary IL-6**	8.6967 ± 12.9693	6.4845 ± 6.9939	0.338
**Serum IL-6**	5.250 ± 9.4003	0.2100 ± 0.42323	<0.001
**Salivary IL-10**	1.9150 ± 3.1140	1.4365 ± 1.0244	0.304
**Serum IL-10**	2.8665 ± 1.0448	1.3880 ± 0.1503	<0.001

A1AT, *α*1-antitrypsin; RA, rheumatoid arthritis; HC, healthy controls; IL, interleukin. Values are presented as the mean ± standard deviation.

**Table 3:** Receiver operating characteristic curve analysis of α1-antitrypsin in saliva.

Outcomes	AUC (95% CI)	*p*-Value	Cut-Off (ng/mL)	Sensitivity	Specificity	LR
*α*1-antitrypsin in saliva	0.7135 (0.5741–0.8529)	0.0134	1689.0	0.62	0.73	2.31

AUC, area under the curve; CI, confidence interval.

**Table 4:** Salivary and serum levels of A1AT, IL-6, IL-1*β*, and IL-10 in patients with seronegative RA and healthy control.

Average Concentration of Studied Cytokines	Seropositive RA	Seronegative RA	HC	*p*-Value (SPRA vs. HC)	*p*-Value (SNRA vs. HC)
**Salivary A1AT (ng/mL)**	2279.78 ± 1619.78	2715.32 ± 2535.79	1579.06 ± 1068.06	0.044	0.121
**Serum A1AT (mg/mL)**	1.7575 ± 5.8905	1.8793 ± 4.4631	1.5932 ± 3.7173	0.180	0.054
**Salivary IL-1*β***	537.34 ± 727.79	551.57 ± 937.95	488.25 ± 379.03	0.723	0.808
**Serum IL-1*β***	1.5460 ± 0.1579	1.5607 ± 0.1647	1.5780 ± 0.1388	0.416	0.745
**Salivary IL-6**	8.7567 ± 14.6085	8.5167 ± 6.1728	6.4845 ± 6.9939	0.400	0.370
**Serum IL-6**	5.2533 ± 5.3263	5.2400 ± 16.8295	0.2100 ± 0.42323	<0.001	0.267
**Salivary IL-10**	1.6487 ± 2.9711	2.7140 ± 3.4943	1.4365 ± 1.0244	0.672	0.189
**Serum IL-10**	3.3182 ± 0.7851	1.5113 ± 0.1998	1.3880 ± 0.1503	<0.001	0.056

Values are presented as the mean ± standard deviation; Fisher’s exact test. A1AT, *α*1-antitrypsin; RA, rheumatoid arthritis; HC, healthy controls; SPRA, seropositive RA; SNRA, seronegative RA; IL, interleukin.

## Data Availability

No new data were created or analyzed in this study. Data sharing is not applicable to this article.
